# Constitutive androstane receptor 1 is constitutively bound to chromatin and ‘primed’ for transactivation in hepatocytes[Fn FN4]

**DOI:** 10.1124/mol.118.113555

**Published:** 2019-01

**Authors:** Michael McMahon, Shaohong Ding, Lourdes Acosta Jimenez, Remi Terranova, Marie-Apolline Gerard, Antonio Vitobello, Jonathan Moggs, Colin J. Henderson, C. Roland Wolf

**Affiliations:** School of Medicine, Jacqui Wood Cancer Centre, Ninewells Hospital and Medical School, University of Dundee, Dundee, United Kingdom (M.M., S.D., L.A.J., C.J.H., C.R.W.) and Preclinical Safety, Translational Medicine, Novartis Institutes for BioMedical Research, Basel, Switzerland (R.T., M.-A.G., A.V., J.M.)

## Abstract

The constitutive androstane receptor (CAR) is a xenobiotic sensor expressed in hepatocytes that activates genes involved in drug metabolism, lipid homeostasis, and cell proliferation. Much progress has been made in understanding the mechanism of activation of human CAR by drugs and xenobiotics. However, many aspects of the activation pathway remain to be elucidated. In this report, we have used viral constructs to express human CAR, its splice variants, and mutant CAR forms in hepatocytes from *Car^−/−^* mice in vitro and in vivo. We demonstrate CAR expression rescued the ability of *Car^−/−^* hepatocytes to respond to a wide range of CAR activators including phenobarbital. Additionally, two major splice isoforms of human CAR, CAR2 and CAR3, were inactive with almost all the agents tested. In contrast to the current model of CAR activation, ectopic CAR1 is constitutively localized in the nucleus and is loaded onto *Cyp2b10* gene in the absence of an inducing agent. In studies to elucidate the role of threonine T38 in CAR regulation, we found that the T38D mutant was inactive even in the presence of CAR activators. However, the T38A mutant was activated by CAR inducers, showing that T38 is not essential for CAR activation. Also, using the inhibitor erlotinib, we could not confirm a role for the epidermal growth factor receptor in CAR regulation. Our data suggest that CAR is constitutively bound to gene regulatory regions and is regulated by exogenous agents through a mechanism which involves protein phosphorylation in the nucleus.

## Introduction

The Constitutive Androstane Receptor (CAR) is a member of the ligand-activated superfamily of nuclear receptor transcription factors (Kobayashi et al., 2015). Early work demonstrated that it was the transcription factor responsible for the induction of hepatic drug metabolising enzymes in response to phenobarbital (PB) treatment (Dumas et al., 1994; Forman et al., 1998; Honkakoski et al., 1998). CAR is now recognized as a key factor in the reprogramming of hepatic gene expression on exposure to a wide range of drugs and environmental chemicals, involving not only the induction of many drug metabolising enzymes and drug transporters, but also proteins involved in lipid, glucose and energy homeostasis, as well as cell proliferation (Shah et al., 2014; Kobayashi et al., 2015). CAR thus substantially regulates liver physiology.

Relatively few activators of human CAR have been reported (Dring et al., 2010; Lynch et al., 2015), which can in part be ascribed to species differences between human CAR and its rodent counterparts (Omiecinski et al., 2011), both in ligand specificity as well as mRNA alternative splicing. In addition to canonical CAR1, at least two additional isoforms, CAR2 and CAR3, are reported to be functional in man (Auerbach et al., 2003). Furthermore, many CAR activators also activate the pregnane X receptor (PXR) resulting in induction of the same proteins. These complexities have been partially overcome by the development of mouse lines humanized for CAR, for example on a *Pxr* null background (Scheer et al., 2008). Although informative, these in vivo systems are not ideally suited to the screening of large numbers of compounds. This has galvanised efforts to establish immortalized in vitro and cell-based reporter assays for CAR. However, such assays are limited as they are unable to detect some bona fide CAR activators, including PB, due to the requirement for hepatocytes to retain differentiated functions.

There has been significant progress in understanding the mechanisms of CAR activation by exogenous agents (Negishi, 2017). CAR can be activated by either direct ligand binding by 6-(4-chlorophenyl)imidazo[2,1-*β*][1,3]thiazole-5-carbaldehyde-O-(3,4-dichlorobenzyl)oxime (CITCO) (Maglich et al., 2003) or indirectly by PB. In the currently accepted model for CAR activation, (Shizu et al., 2017), the inactive transcription factor is a cytoplasmic homodimer phosphorylated at T38. The homodimer is stabilized by signaling through the Epidermal Growth Factor Receptor (EGFR) pathway. PB exposure inhibits the effects of EGFR and destabilizes the complex, converting CAR into a monomer, exposing phospho-T38 which is then de-phosphorylated by the Protein Phosphatase 2 (PP2A)/Receptor of Activated Protein Kinase C 1 (RACK1) phosphatase complex, resulting in CAR translocation to the nucleus and transcriptional activation. Direct-binding CAR activators, such as CITCO, induce CAR monomerisation as a consequence of binding to the CAR protein (Yang and Wang, 2014; Negishi, 2017).

Although data supporting this mechanism of CAR regulation is quite compelling, a number of questions remain. Is nuclear CAR constitutively active or does it require further activation? There is evidence that nuclear translocation alone is insufficient for CAR activation and other as yet to be defined signaling processes are required for activation (Rencurel et al., 2005; Koike et al., 2007; Ohno et al., 2014). Moreover, how does PB inhibit EGFR? Is EGFR inhibition a pre-requisite for all indirect CAR activators? Do other EGFR inhibitors activate CAR? Also, does the proposed mechanism apply to the putatively active human CAR splice variants?

To gain further insight into the mechanism of gene regulation by CAR we have used a model hepatocyte system involving *Car^−/−^* mice transduced in vitro or in vivo with viral vectors expressing native or mutant human CAR proteins. We validate the model and show that the two major non-canonical CAR splice isoforms, CAR2 and CAR3, are cytoplasmic and functionally inactive. Importantly, we also provide evidence that questions the current model of CAR activation. Specifically, we find that CAR1 isoform is constitutively present in the hepatocyte nucleus and bound to regulatory regions of the CAR-responsive gene, *Cyp2b10*. We also provide evidence that CAR activators must further modify nuclear CAR to induce gene expression in a manner which does not involve the phosphorylation of T38, as the T38A mutant still required activation. The EGFR inhibitor erlotinib did not activate CAR, questioning the role of EGFR in regulating CAR activation.

## Materials and Methods

### 

#### Chemicals, Antibodies, and Recombinant Proteins.

Unless otherwise stated, all chemicals were purchased from Sigma-Aldrich (Dorset, UK). CITCO was obtained from TOCRIS Bioscience (Abingdon, Oxfordshire, UK). Okadaic acid (OA) and erlotinib were both from Calbiochem (Merck, Watford, Herts, UK). Mouse anti-FLAG antibody (clone M2) was from Sigma. Rabbit anti-FLAG antibodies conjugated to Alexa Fluor 555 were from Cell Signaling technology (New England Biolabs, Hitchin, Herts, UK). Rabbit antibodies against: rat CYP2B1, CYP3A1 and CYP4A1 have been previously described, and have been shown to cross-react specifically with the corresponding murine P450 enzymes (Forrester et al., 1992; Henderson et al., 2003; Scheer et al., 2014). Antibodies against glucose regulated protein 78 (GRP78) were from Abcam (Milton, Cambridge, UK). Recombinant Cyp2b10 and Cyp3a11 were prepared as previously described (McLaughlin et al., 2008).

#### Animal Husbandry and Reconstitution with Human CAR Proteins.

All animal work was carried out in accordance with the UK government’s Animal Scientific Procedures Act (1986). All animal studies were approved by the Ethical Review Committee, University of Dundee. Efforts were made to limit the number of subjects and minimize animal suffering according to the principles of reduction, replacement and refinement in experimental design.

Unless otherwise stated, female mice on a C57BL/6NTac background, aged between 8 and 16 weeks, were used in these studies. Homozygous *Car^−/−^* mice, and mice humanized for PXR and/or CAR have already been described, as have the relevant genotyping protocols (Scheer et al., 2008). We also used *Car^−/−^* mice containing a CYP2B6-LacZ reporter gene (construction of the CYP2B6-LacZ reporter mouse is described In Supplemental Information). Finally, we used double-humanized *hCAR*/*hPXR* mice containing the CYP2B6-LacZ reporter gene.

All animals were bred at the University of Dundee, School of Medicine’s Animal Unit and were housed in Tecniplast Sealsave microisolator cages containing Eco-Pure chip7D (Datesand Group, Manchester, UK) for bedding. Cages also contained red polycarbonate huts (Datesand Group). Mice were segregated by gender and housed with siblings. Food (RM1 pelleted diet supplied by Special Diet Services Ltd, Stepfield, Witham, Essex, UK) and drinking water (taken from the local supply) were available ad libitum. Light cycles were on a 12:12 hour light:dark cycle with the light phase starting at 0600 hour. Temperature and relative humidity were maintained between 21 and 23°C, and 45% and 65%, respectively. To reconstitute *Car^−/−^* mice with human CAR proteins, mice were injected with 2 × 10^9^ infectious units of the appropriate adenovirus through the tail-vein. Mice were dosed with chemicals not less than 24 hour after virus injection to allow time for transgene expression. Mice were not fasted and all interventions were performed during the light cycle.

#### Culture and Treatment of Mouse Primary Hepatocytes.

Mouse primary hepatocytes were isolated from female *Car^−/−^* CYP2B6-LacZ reporter mice by a two-step collagenase perfusion method and cultured, as previously described (Klaunig et al., 1981a,b), with modification. Briefly, after perfusion with buffer (137 mM NaCl, 7 mM KCl, 0.7 mM Na_2_HPO_4_, 10 mM HEPES, and 5.1 mM CaCl_2_, pH7.65) containing type I collagenase (from *Clostridium histolyticum* type IV; Sigma), and filtration through a 70 *μ*m cell strainer, hepatocytes were suspended in William’s medium E supplemented with 10% (v/v) fetal bovine serum, 2 mM L-glutamine and 30 mM pyruvate and were allowed to adhere to six-well (1 × 10^6^ cells) or 12-well plates (4 × 10^5^ cells) for 4 hours in a CO_2_ incubator at 37°C. Plates had been pre-coated with 12.5 *μ*g/cm^2^ rat-tail collagen type I (Gibco, ThermoFisher Scientific, Perth, UK). Subsequently, the William’s medium was replaced with serum-free HepatoZYME-SFM containing 5 *μ*g/ml rat-tail collagen type I and adenovirus particles. The multiplicity of infection (MOI) for each viral preparation (AdCAR1: 5; AdCAR2: 12; AdCAR3: 12; AdCAR1T38A: 25; AdCAR1T38D: 20; empty adenovirus: 20) was chosen to ensure equal expression of the various heterologous CAR proteins. Post-infection (24 hours), hepatocytes were shifted to adenovirus- and collagen-free HepatoZYME-SFM medium containing test compounds or vehicle control. After 24 hour of chemical treatment, LacZ activity was measured using the Galacto-light Plus system (ThermoFisher Scientific), according to the manufacturer’s instructions.

#### Immunofluorescent Detection of FLAG-Tagged CAR.

To visualize FLAG-tagged CAR protein, mouse primary hepatocytes isolated from *Car^−/−^* mice were cultured in six-well plates containing a glass coverslip coated with rat-tail type I collagen (Gibco), infected with adenoviral particles expressing FLAG-tagged CAR, and exposed to chemicals as described above. Post-treatment, coverslips were washed twice at room temperature with PBS and fixed for 20 minute in 4% (w/v) paraformaldehyde. After blocking and permeabilizing the cells for 1 hour at room-temperature in a solution of 5% (v/v) fetal bovine serum and 0.3% (v/v) Triton-X-100 in PBS, they were stained overnight at 4°C with 10 *μ*g/ml of AlexaFluor 555-conjugated rabbit anti-FLAG in PBS. Coverslips were subsequently rinsed three times in PBS with 0.1% (v/v) Triton X-100 at room temperature and mounted with Vectashield mounting medium with 4′,6-diamidino-2-phenylindole (Vectashield). Images were captured by confocal microscopy using the Leica S5 microscope equipped with a 40× NA 1.25 PlanApochromat oil-immersion objective. Fluorescent signals of AlexaFluor-555 (excitation 543 nm using a HeNe1 laser) and 4′,6-diamidino-2-phenylindole (excitation 405 nm using a UV laser) were detected using PMTs with spectral ranges of 551–608 nm and 427–485 nm, respectively. Images were rendered in ImageJ.

#### Quantitative PCR (qPCR).

Quantitative PCR assessment of hCAR-FLAG chromatin enrichment at the *Cyp2b10* locus was performed using Fast SYBR Green Master Mix (Applied Biosystems (ThermoFisher Scientific, Perth, UK)), according to the manufacturer’s instructions. *Cyp2b10* primers were designed to encompass accessible regulatory regions upstream of the *Cyp2b10* transcriptional start site (TSS). Additional *Cyp2b10* intragenic as well as control genomic regions (*homeobox A9* (*Hoxa9)*, *glyceraldehyde-3-phosphate dehydrogenase* (*Gapdh*)) were selected as negative control regions. All primers are listed in Supplemental Table S1. The specificity and quality of amplification was controlled for each of the PCR primer sets used in this study (data not shown). To calculate enrichment level, the amount of qPCR product in the immunoprecipitate was expressed as a percentage of the amount of product in the input DNA. Results were normalized using one of the negative control primer set, *Gapdh*.

Additional Experimental Procedures, including construction of recombinant adenovirus’, generation of CYP2B6-LacZ reporter mouse, in situ *β*-galactosidase staining, preparation of protein extracts and immunoblotting, and Chromatin Immunoprecipitation (ChIP), can be found in Supplemental Text.

## Results

### 

#### Hepatic Expression of Functionally Active Human CAR Isoforms in *CAR^−/−^* Mice.

**Fig. 1.**
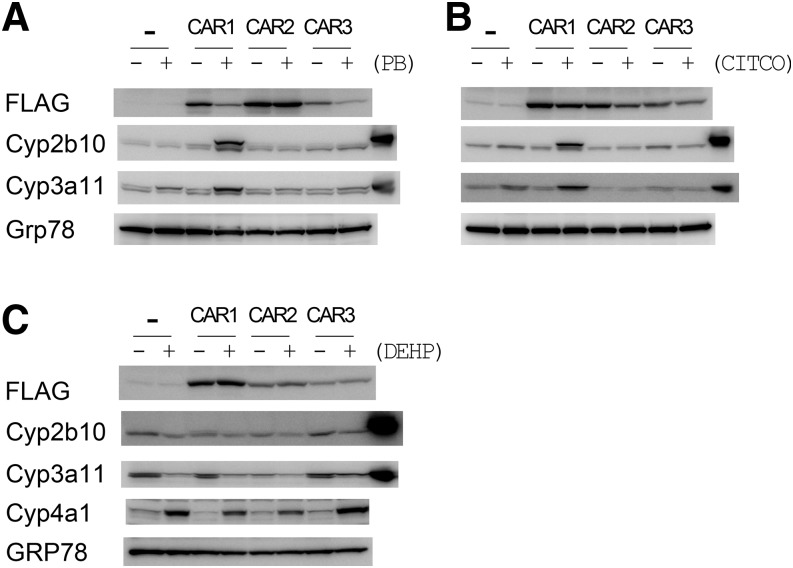
Expression of human CAR isoforms and P450 induction in *Car^−/−^* mice. *Car* knock-out mice were injected on day 0 with either empty adenovirus (-) or adenovirus expressing FLAG-tagged human CAR1, CAR2, or CAR3. The mice were subsequently dosed with 80 mg/kg PB ip (A), 50 mg/kg CITCO ip (B) or (C) DEHP at 150 mg/kg ip on each of days 1, 2, and 3. Animals were sacrificed on day 4. Liver microsomes from each animal were pooled (*n* = 3) and equal amounts of protein blotted for CAR target proteins (Cyp2b10 and Cyp3a11). The final track in the Cyp2b10 and Cyp3a11 blots contains positive standards (liver microsomes prepared from PB (Cyp2b10) or rifampicin (Cyp3a11) treated wild-type mice). Grp78 was used as a loading control. Whole-cell lysates were blotted for FLAG-tagged CAR.

Analysis of liver microsomes from *Car^−/−^* mice infected with either empty control adenovirus or adenovirus’ expressing CAR isoforms showed that all the human CAR isoforms were expressed (Fig. 1A). None of the isoforms displayed constitutive activity as assessed by the expression of two CAR target genes, *Cyp2b10* and *Cyp3a11* (Fig. 1A). Moreover, in agreement with the literature (Jinno et al., 2004), CAR1, but not CAR2 or CAR3, was activated by the indirect acting PB (Fig. 1A). CITCO, a direct-acting CAR ligand, induced hepatic *Cyp2b10* or *Cyp3a11* also only in mice expressing CAR1 (Fig. 1B), despite being reported to activate all CAR isoforms on the basis of in vitro transactivation assays (Auerbach et al., 2005). These data demonstrate that human CAR variants can be expressed in mouse liver using a viral delivery system and that CAR1 responds to both direct and indirect CAR activators.

**Fig. 2.**
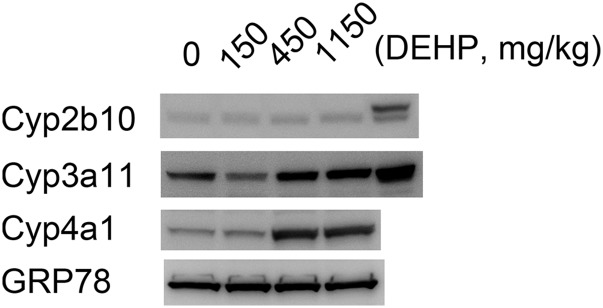
DEHP does not activate human CAR isoforms. hCAR/hPXR double-humanized mice were treated with the indicated doses of DEHP (mg/kg) on each of days 1, 2, and 3. Animals were sacrificed on day 4. Liver microsomes from each animal were pooled (*n* = 3) and equal amounts of protein blotted for CAR target proteins (Cyp2b10 and Cyp3a11). The final track in the Cyp2b10 and Cyp3a11 blots contains positive standards (liver microsomes prepared from PB (Cyp2b10) or rifampicin (Cyp3a11) treated wild-type mice).

To establish whether CAR2 or CAR3 were active toward other inducers, we tested whether the Peroxisome Proliferator-Activated Receptor *α* (PPAR*α*) agonist diethylhexyl phthalate (DEHP – reported to be a specific activator of CAR2 (DeKeyser et al., 2009) would induce *Cyp2b10* and *Cyp3a11*. Although DEHP administration induced the expression of PPAR*α*-regulated *Cyp4a11*, no induction of *Cyp2b10* and *Cyp3a11* was observed (Fig. 1C). To investigate this further, we treated mice humanized for CAR, (expressing all three CAR variants (Scheer et al., 2008)), with various doses of DEHP. In agreement with our reconstitution system, DEHP activated PPAR*α* (*Cyp4a*) but not CAR (*Cyp2b10*) (Fig. 2).

#### Human CAR Activation in Mouse Primary Hepatocytes.

**Fig. 3.**
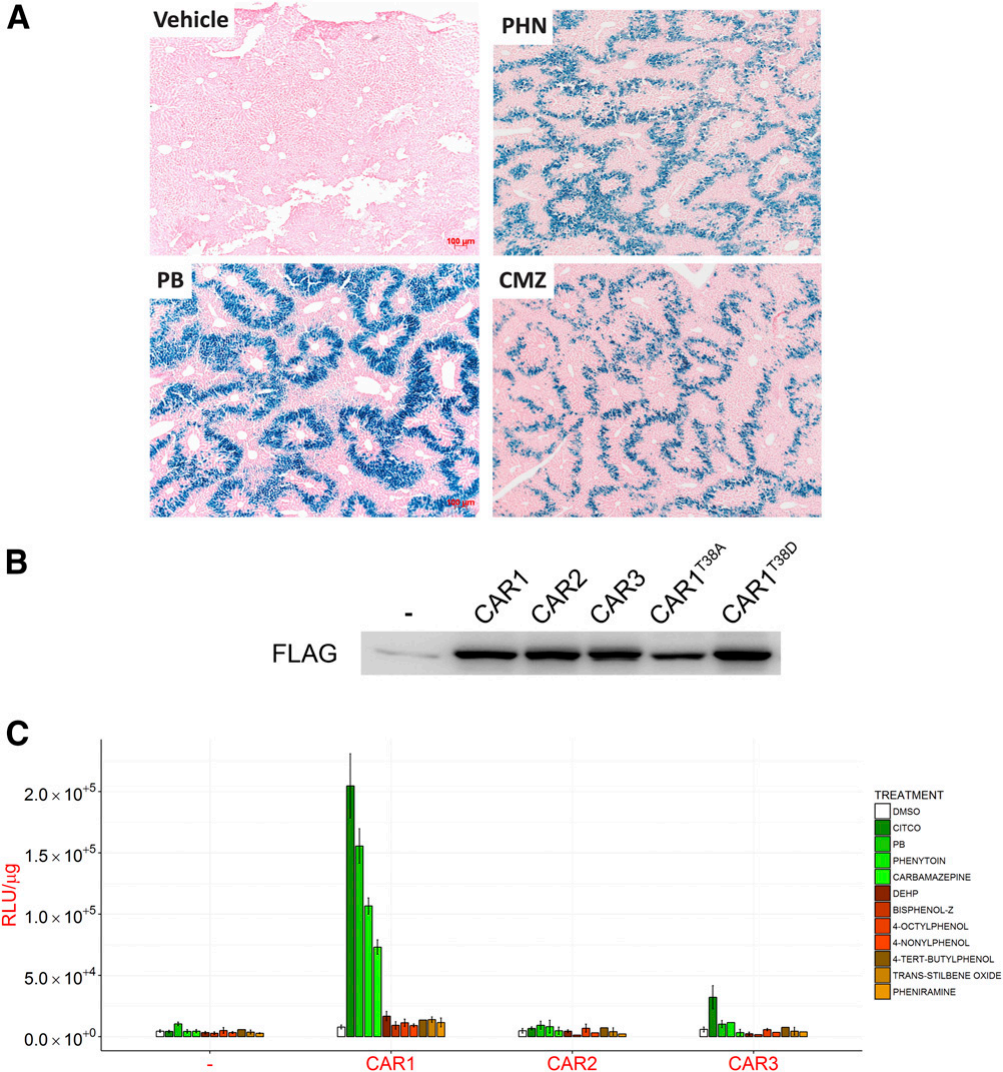
Human CAR activity can be measured ex vivo in primary mouse hepatocytes. (A) Male C57BL/6 mice from a transgenic reporter model where murine Car and Pxr are replaced with their human counterparts CAR and PXR, and bearing a CYP2B6-LacZ reporter transgene, were treated with phosphate-buffered saline (PBS; Vehicle), phenobarbital (PB; 80 mg/kg), phenytoin (PHN; 50 mg/kg) or carbamazepine (CMZ; 250 mg/kg) intraperitoneally (ip) at 5 ml/kg, daily for 3 days (*n* = 3). Representative liver sections stained for LacZ are shown. (B and C) Hepatocytes from *Car^−/−^* CYP2B6-LacZ reporter mice were reconstituted with empty control adenovirus (-) or adenoviruses expressing human CAR isoforms or mutants thereof. (B) Expression of FLAG-tagged proteins was determined by immunoblot. (C) CYP2B6-LacZ reporter activity (RLU/*μ*g protein) was measured 24 hour after exposure to the indicated chemicals. All chemicals were used at a final concentration of 10 *μ*M except PB (2.5 mM), phenytoin (20 *μ*M), carbamazepine (25 *μ*M), DEHP (50 *μ*M), and *trans*-stilbene oxide (50 *μ*M). Data represent the mean ± S.D. of three separate experiments.

To study the mechanism of CAR activation we expressed human CAR proteins in freshly isolated *Car^−/−^* hepatocytes. For in vitro measurement of CAR activity, we used hepatocytes from a *Car^−/−^* mouse line that additionally carried a CAR-responsive human CYP2B6-lacZ reporter. This CYP2B6-LacZ reporter was highly inducible in mice treated with three CAR activators (PB, phenytoin, and carbamazepine (Fig. 3A)), but was not inducible in hepatocytes from CAR null mice (unpublished), By titrating the multiplicity of infection for each adenovirus preparation equivalent expression of the three CAR splice variants was obtained (Fig. 3B). None of the three CAR isoforms had any basal activity, as assessed using the CYP2B6 reporter (Fig. 3C). On treatment of the CAR-expressing hepatocytes with PB or CITCO, a robust increase in LacZ expression was observed in CAR1 expressing cells, whereas DEHP failed to induce LacZ activity in cells expressing any of the CAR isoforms (Fig. 3C). We next studied eight additional chemicals previously investigated for their capacity to activate some or all of the human CAR isoforms. These included four phthalates (4-nonylphenol, reported to solely activate CAR1 (DeKeyser et al., 2011) or CAR3 (Dring et al., 2010), 4-octylphenol, 4-*tert*-butylphenol (CAR2 activator (Dring et al., 2010)) and bisphenol-Z (CAR3 activator (Dring et al., 2010)); *trans*-stilbene oxide (CAR2 activator (Dring et al., 2010)); two anticonvulsants, phenytoin and carbamazepine both of which induce *CYP2B6* expression in human hepatocytes (Faucette et al., 2007; Hariparsad et al., 2008); and the antihistamine pheniramine, a putative CAR3-specific ligand (Dring et al., 2010). Consistent with our in vivo experiments (Fig. 3A), only two – PHN and CMZ – activated CAR1 (Fig. 3C). CAR2 and CAR3 were essentially inactive for all chemicals tested apart from a very slight activation of CAR3 by CITCO (Fig. 3B). These data suggest that of the three tested isoforms, CAR1 is the only functional isoform of pharmacological significance.

#### CAR1 Is Constitutively Localized in the Nucleus.

**Fig. 4.**
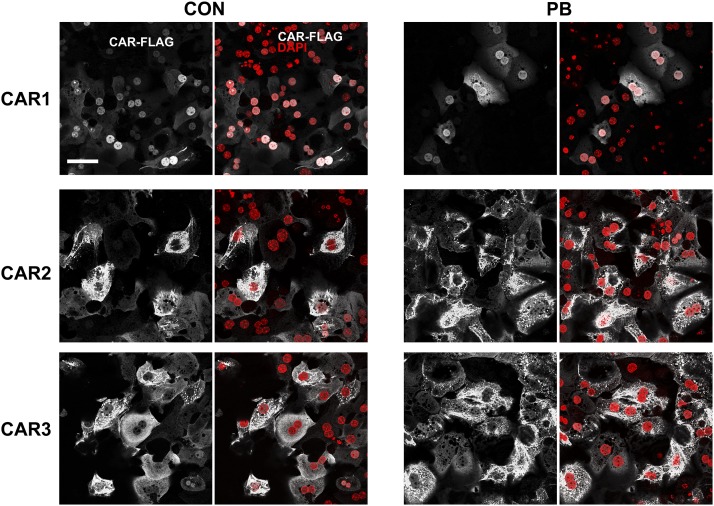
CAR1 is primarily nuclear in both control and PB-treated primary mouse hepatocytes. The photomicrographs depict the location of FLAG-tagged CAR isoforms in mouse primary hepatocytes 24 hour after treatment with vehicle (PBS) or 2.5 mM PB. DAPI was used as a nuclear counterstain. Scale bar, 50 *μ*m.

According to current models, CAR is retained in the cytoplasm until activated by inducing agents (Yang and Wang, 2014). To investigate this, we carried out immunocytochemistry using a FLAG antibody on cells infected with adenoviral CAR1. Surprisingly, CAR1 was almost exclusively nuclear in the majority of hepatocytes in the absence of a CAR activator (Fig. 4). On the basis of DAPI staining, there was some suggestion that the transcription factor is specifically excluded from nucleoli (Fig. 4). In a small percentage of cells, CAR1 was found outside the nucleus, near the plasma membrane and/or in punctate structures in the cytoplasm (Supplemental Fig. S1). Although only a very small proportion of all CAR1-expressing cells, this population is prominent as the CAR1 signal was more intense in this population than in cells displaying nuclear CAR1. In contrast, CAR2 and CAR3 isoforms were only located in the cytoplasm of all cells, with a punctate distribution (Fig. 4).

Upon PB treatment, CAR1 was located exclusively in the nucleus with no minor population exhibiting cytoplasmic localization observed (Fig. 4), suggesting that PB induces CAR1 translocation from the cytoplasm to the nucleus in these cells. Notably, the cytoplasmic location of both CAR2 and CAR3 remained unaffected by exposure to PB, consistent with their inability to induce gene expression (Fig. 4).

**Fig. 5.**
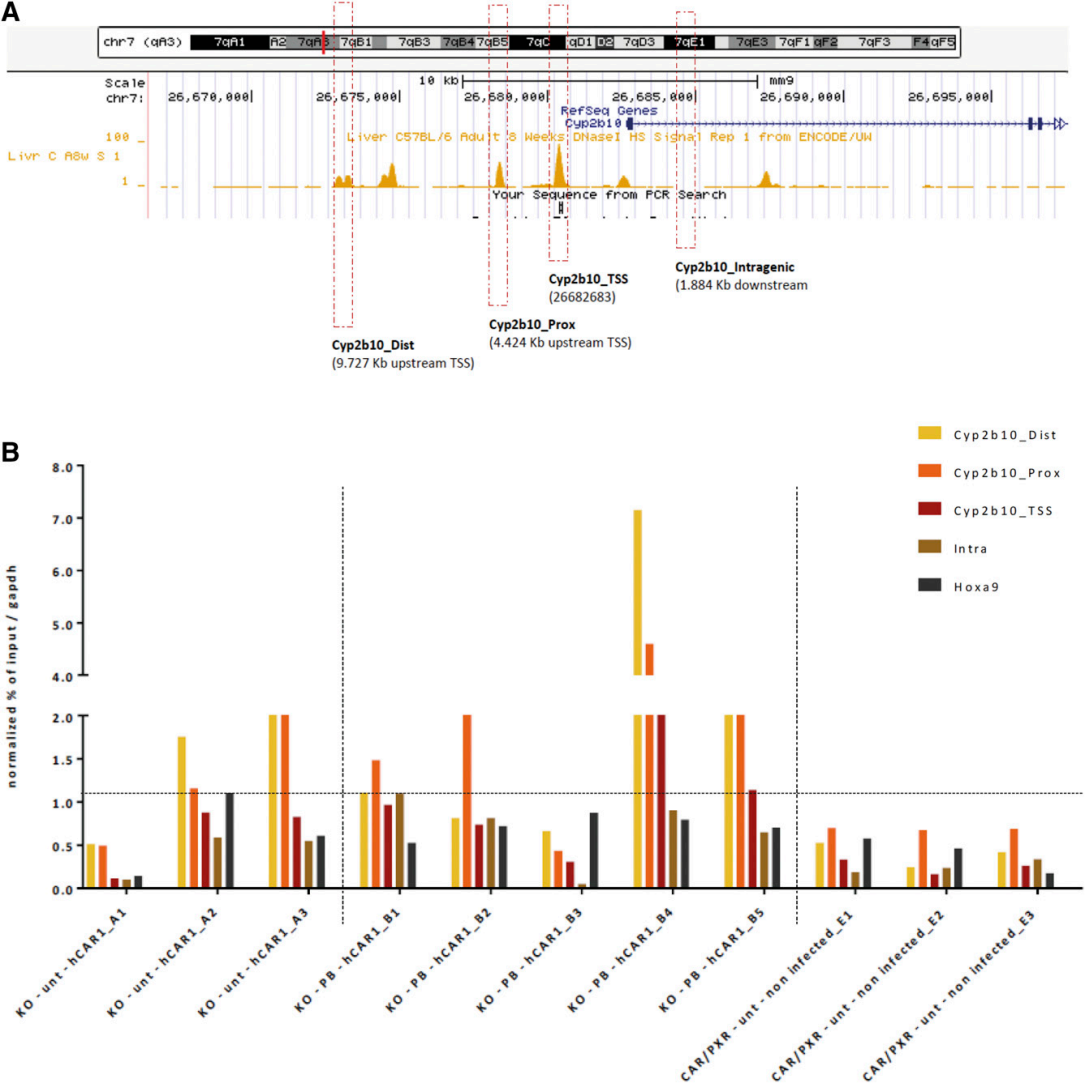
CAR1 is pre-bound at the *Cyp2b10* promoter in untreated hepatocytes. (A) Depiction of the location of PCR amplicons in and upstream of the *Cyp2b10* gene that were chosen for use in ChIP analysis of chromatin occupancy by FLAG-tagged CAR1. The track displays publically-available, DNase I hypersensitivity data for C57BL/6 liver (ENCODE ENCSR000CNI, replicate 1) and highlights that the chosen amplicons fall within DNase I-sensitive regions of chromatin. (B) Enrichment of *Cyp2b10* proximal and distal amplicons, and negative control amplicons (*Cyp2b10* intragenic region, *Hoxa9*, *Gapdh*) in anti-FLAG-immunoprecipitates from untreated mice reconstituted with CAR1 (Group A), PB-treated mice reconstituted with CAR1 (positive control samples – Group B), or untreated mice reconstituted with empty virus (negative control samples – Group E). Enrichment data are presented for individual mice and are normalized to the *Gapdh* signal. The horizontal dashed-line represents the limit of detection for enrichment.

On the basis of its nuclear localization, we considered the possibility that CAR1 might be constitutively bound to chromatin at regulatory genomic binding sites, such as the *Cyp2b10* promoter and regulatory regions, in the absence of an activator. Accordingly, we transfected *Car^−/−^* mice with FLAG-tagged CAR1 and the mice were either left untreated (Supplemental Table S2 – Group A) or treated with PB for 3 days (Supplemental Table S2 – Group B). As negative controls, we transduced the livers of *Car^−/−^ Pxr^−/−^* double humanized mice with empty adenovirus (Supplemental Table S2 – Group E). ChIP experiments with an anti-FLAG antibody were used to detect the presence of chromatin-bound FLAG-tagged CAR1 in these liver samples. PCR primers were designed to encompass accessible regulatory regions (DNase I-sensitive and bound by transcription regulators) flanking the *Cyp2b10* transcriptional start site (TSS) (Fig. 5A; Supplemental Table S1). ChIP-qPCR evaluations of CAR1-FLAG binding in individual experimental samples demonstrated CAR1 enrichment in the absence of PB treatment in two out of three animals tested (A3 and A2), with apparent preferential enrichment at the proximal and distal *Cyp2b10* regulatory regions (Fig. 5B). In four out of five individual experimental samples from PB-treated animals, (PB treated, B4 > B5 > B2 > B1) CAR1 was enriched in at least at one of the *Cyp2b10* regulatory regions (Fig. 5B). Importantly, no enrichment was detected in any of the negative control samples (Fig. 5B). The limit of detection for enrichment is set by the signal detected using negative controls regions such as Intragenic *Cyp2b10* or *Hoxa9* (dotted line). Altogether, these experiments demonstrate the constitutive chromatin binding of hCAR1 at *Cyp2b10* regulatory regions, in the absence of PB treatment.

#### Studies on the Role of T38 in Car Activation.

**Fig. 6.**
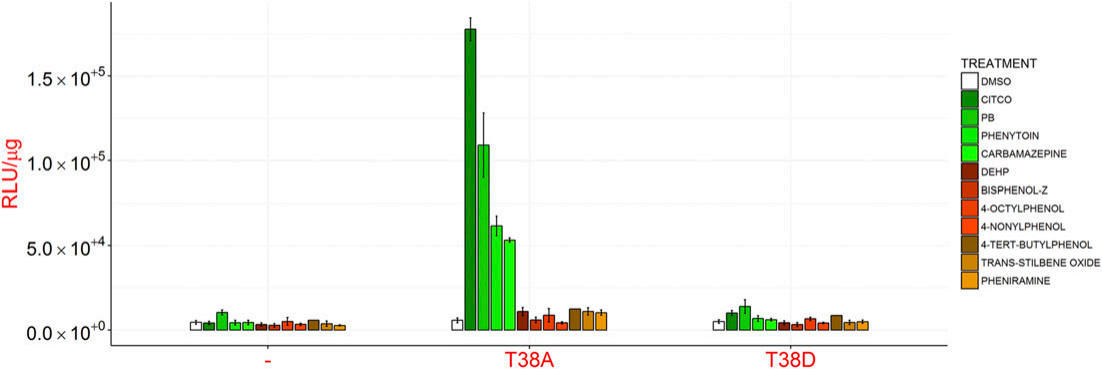
CAR1^T38A^ is not constitutively active. Hepatocytes from *Car^−/−^* CYP2B6-LacZ reporter mice were reconstituted with empty control adenovirus (-) or adenoviruses expressing human CAR1^T38A^ or CAR1^T38D^. CYP2B6 reporter activity (RLU/*μ*g protein) was measured 24 hour after exposure to the indicated chemicals. All chemicals were used at a final concentration of 10 *μ*M except PB (2.5 mM), phenytoin (20 *μ*M), carbamazepine (25 *μ*M), DEHP (50 *μ*M), and *trans*-stilbene oxide (50 *μ*M). Data represent the mean ± S.D. of three separate experiments. Note that these data were collected alongside the data presented in Fig. 3C; the empty control adenovirus data are shared between the two datasets.

**Fig. 7.**
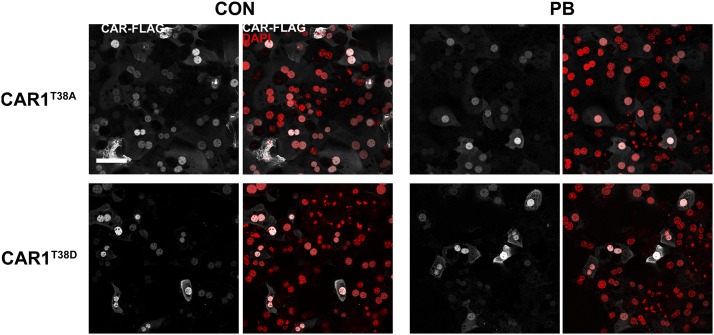
Residue T38 is dispensable for correct CAR1 localization in primary mouse hepatocytes. Photomicrographs depict the location of FLAG-tagged CAR1 bearing amino-acid substitutions at residue T38 in mouse primary hepatocytes 24 hour after treatment with vehicle (PBS) or 2.5 mM PB. DAPI was used as a nuclear counterstain. Scale bar, 50 *μ*m.

The finding that CAR1 was localized at high levels in the nucleus in untreated cells led us to investigate the role of T38 in CAR1 regulation as in the model proposed by Negishi et al. it controls CAR nuclear translocation in the presence of an inducing agent (Negishi, 2017). In contrast to literature reports, expression of CAR1 bearing a non-phosphorylatable alanine residue in place of T38 (T38A) in hepatocytes did not result in induction of CAR responsive genes (Fig. 6). Instead, it was indistinguishable from the wild-type protein in both in terms of inducibility (*cf* Fig. 3B; Fig. 6) and subcellular distribution i.e., predominantly nuclear (Fig. 7) with occasional cytoplasmic staining in untreated cells (Supplemental Fig. S1). These data provide evidence that dephosphorylation of T38 alone is not sufficient to activate CAR1, and also suggest that both indirect and direct-acting compounds are able to activate CAR1 by a mechanism other than promoting T38 dephosphorylation.

To investigate this further we studied the properties of CAR1^T38D^, a mutated form of CAR1 where the negatively-charged aspartic acid mimics phosphorylated T38. This amino-acid change is also predicted to reduce the ability of the nuclear receptor to bind DNA (32). Consistent with the reported central role of this phosphorylation event in CAR function (32), CAR1^T38D^ lacked transcriptional activity even after stimulation by PB or any of the other CAR activators tested (Fig. 6). However, in contrast with previous reports that CAR1^T38D^ is sequestered in the cytoplasm in healthy and stimulated hepatocytes (Mutoh et al., 2009), the subcellular distribution of this form of the protein was indistinguishable from the wild-type protein in control and PB-treated hepatocytes (Fig. 7), including a complex cytoplasmic distribution in a prominent minor subpopulation of control – but not PB-treated – cells (Supplemental Fig. S1).

The above findings suggest that cytoplasmic retention is not a key factor in controlling CAR activity. PB has been reported to promote CAR1 translocation to the nucleus by inhibiting EGFR and consequently promoting de-phosphorylation of T38 (Mutoh et al., 2013). We reasoned that if inhibition of EGFR was necessary and sufficient for activation of CAR1 by PB, then inhibition of EGFR by other drugs should also activate CAR. We found that although treatment of hepatocytes with the EGFR inhibitor erlotinib alone abrogated EGFR signaling (Supplemental Fig. S2A), it did not stimulate CAR transcriptional activity (Supplemental Fig. S2B. Indeed, pre-treatment of hepatocytes with this inhibitor actually antagonized the activation of CAR by PB by a modest but statistically significant extent (Supplemental Fig. S2B). Moreover, we were unable to demonstrate any inhibition of EGFR by PB as determined by loss of Erk1/2 phosphorylation (Supplemental Fig. S2A). These data show that EGFR inhibition per se does not play a major role in controlling CAR1 activation by PB. However, protein phosphorylation does appear important as, consistent with previous reports (34), pre-treatment of hepatocytes with okadaic acid, a PP2A-specific phosphatase inhibitor, reduced CAR1 activity in PB treated cells (Supplemental Fig. S2B).

## Discussion

CAR was first isolated as a key regulator of hepatic drug metabolising enzymes in response both to drugs and environmental chemicals. However, it also controls a number of other important hepatic functions. Understanding CAR regulation and the downstream consequences is of central importance in understanding hepatocyte function and also in predicting drug/drug interactions and pathways of drug and chemical toxicity (Luisier et al., 2014; Yang and Wang, 2014). It could be argued that regulation of CAR in mice differs from that in humans; however, the system used recapitulates the mechanism of gene regulation by known CAR activators and based on our current knowledge, although species differences in ligand specificity and in the downstream genes they regulate have been described, to date no differences in the mechanism of CAR regulation have been reported.

Mechanistic insights into CAR regulation have been pioneered by Negishi (2017). The data presented in this paper extends some of this work and questions certain aspects of the current model. In the first instance, contrary to data obtained using in vitro transactivation assays with transformed non-hepatic cell lines (DeKeyser et al., 2009, 2011; Dring et al., 2010) the splice variants CAR2 and CAR3, which only differ from CAR1 by three and five amino acids respectively (Auerbach et al., 2003), were inactive toward essentially all the compounds tested apart from a very minor activation of CAR3 by CITCO. The reason for this difference between our findings and published data are unclear but probably relates to the different cells used, i.e., between primary hepatocytes and immortalized non-hepatocyte cell lines. It is relevant to note that unlike CAR1, both CAR2 and CAR3 were located in the cytoplasm in the presence and absence of the CAR inducing agent PB, (with the caveat that PB did not activate CAR2 or CAR3). It is interesting to note that DEHP at doses which activated PPAR*α*, were inactive toward all three CAR isoforms) in vivo. These data show that the small amino acid differences in CAR sequence determine its subcellular localization and also explains why the splice variants are essentially inactive.

The finding that the majority of expressed CAR1 was in the nucleus in the absence of chemical activators is in contrast with a number of studies reported in the literature (Yang and Wang, 2014; Negishi, 2017). However, it is consistent with the finding that the vitamin D receptor (Bertrand et al., 2004), the nuclear receptor most closely related to CAR, is also located in the nucleus (Haussler et al., 1998). Indeed, this location is also a characteristic of other unliganded Class II-type nuclear receptors, of which CAR is one (McKenna and O’Malley, 2002).

The direct evidence that endogenous CAR1 is cytoplasmic, i.e., which does not use tagged CAR1 proteins to track CAR1 localization, comes from subcellular fractionation experiments. This approach has its own limitations in that nuclear proteins, including nuclear receptors, have been shown to distribute into the cytosolic fraction on differential centrifugation (Yamamoto, 1985).

Wild-type CAR1 and both of the T38 mutant forms were almost exclusively in the nucleus. If this was due to the over-expression of CAR, a combination of both cytoplasmic and nuclear staining would have been expected – this was not the case. The difference between our findings on CAR localization and those reported in the literature could be due to the expression of the much smaller FLAG- versus GFP-tagged CAR (Guo et al., 2007; Mutoh et al., 2009). In both cases, the expression of CAR was at higher than physiologic levels, which therefore would not explain the difference in the data. Also, if this was the case in our experiments it would not explain why CAR2 and CAR3, when expressed at the same level, were located in the cytoplasm. The nuclear localization of CAR was also supported by ChIP analyses, which showed that CAR1 was constitutively bound to proximal and distal regions of the *Cyp2b10* gene in mouse liver in vivo. Interestingly, CAR1 binding was at novel regions of the gene and enrichment was not observed at the *Cyp2b10* transcription start site containing the Phenobarbital-responsive Element Module (PBREM). Preliminary data using *DNaseI* fingerprinting on in vivo samples showed that the endogenous Car1 protein is bound constitutively to the *Cyp2b10* promoter (Terranova, et al., unpublished data).

In a small number of cells CAR1 expression was cytoplasmic and it is possible that these are cells in which CAR1 becomes activated by exogenous chemicals in a manner which is consistent with the findings of Negishi et al. Indeed, cytoplasmic CAR1 staining was no longer detected on the administration of the CAR activator phenobarbital. However, it is unlikely that this small number of cells could account for the transactivation responses observed. Although it should be noted that there may not be a direct relationship between the observed level of nuclear receptor and the degree of transactivation. Also, if this were the case, it would not explain why nuclear CAR1 was not constitutively active. It will be interesting to establish what distinguishes these hepatocytes from the rest of the population. It should be noted that Negishi and co-workers have recently also shown constitutive CAR 1 nuclear localization (Shizu et al., 2017) and independent of differences in the reports our data demonstrates that the sequestering of CAR in the cytoplasm is not a key factor in supressing its activity.

A major advance in understanding the mechanism of CAR activation was the identification of T38 as a critical amino acid in this process. The most recent model proposed that phospho-T38 retains CAR in the cytoplasm in a dimeric form which is converted to a monomer on exposure of hepatocytes to either a direct ligand or indirect-acting activators. This results in the removal of the phosphate group which facilitates nuclear translocation (Mutoh et al., 2009; Negishi, 2017). However, our data demonstrates that T38 is dispensable for CAR1 activation as the CAR1^T38A^ mutant was not constitutively active and required an exogenous activator to become functional. Our finding that the T38D mutant is inactive suggests that T38 is an important structural amino acid.

A major unifying mechanism of nuclear receptor function is that activation, for example by a ligand, increases transcriptional activity by increasing the affinity for nuclear coactivators by ligand-induced conformational changes in the receptors (McKenna and O’Malley, 2002). It is now also clear that these interactions can be modulated by signal transduction pathways that lead to phosphorylation of either coactivator complexes or nuclear receptors themselves (Gronemeyer et al., 2004). Our studies suggest that PB promotes the interaction of CAR with one-or-more coactivator complexes to activate transcription. Consistent with this hypothesis was our finding and those of others that the phosphatase inhibitor okadaic acid inhibited the action of PB (Honkakoski and Negishi, 1998). Moreover, an increased interaction with a coactivator complex may explain the redistribution of CAR1 in hepatocytes where cytoplasmic staining was observed (Negishi, 2017). Based on previous studies, it is interesting to speculate that nuclear AMPK may be involved (Rencurel et al., 2005; Kodiha et al., 2007).

A number of compounds activate CAR by an indirect mechanism and it has been reported that in the case of PB this involves inhibition of EGFR signaling (Mutoh et al., 2013). In our study PB did not inhibit EGFR, nor did the EGFR inhibitor Erlotinib induce CAR-mediated gene expression. These data argue against a role for EGFR in CAR transcriptional activation by phenobarbital and possibly other indirect activators. Recently, Shizu et al. reported that erlotinib induced CAR-mediated *Cyp2b10* expression, albeit only threefold relative to a 15-fold induction with PB (Shizu et al., 2017). Our data suggest that signaling through MEK/ERK is not involved in CAR activation. However, this could be further investigated through the use of specific inhibitors and activators of this pathway.

In summary, based on the data above, our current hypothesis is that CAR is constitutively bound to CAR-responsive genes and is activated as a consequence of a phosphorylation event of itself or co-activator proteins. The kinases and phosphatases involved in this process remain to be identified but AMPK may be involved. The mechanism of CAR1 activation in the nucleus remains an important area for research; in this study, we have exemplified a powerful experimental approach for this purpose.

## Abbreviations

CAR: Constitutive Androstane Receptor
ChIP: Chromatin-immunoprecipitation
CITCO: 6-(4-chlorophenyl)imidazo[2,1-*β*][1,3]thiazole-5-carbaldehyde-O-(3,4-dichlorobenzyl)oxime
DEHP: Diethylhexyl Phthalate
EGFR: Epidermal Growth Factor Receptor
ERK1/2: extracellular signal-regulated kinase 1/2
*β* Gal: *β*-galactosidase
Gapdh: glyceraldehyde-3-phosphate dehydrogenase
Hoxa9: homeobox A9
MOI: multiplicity of infection
OA: Okadaic acid
PB: Phenobarbital
PP2A: Protein Phosphatase 2
PPAR *α*: Peroxisome Proliferator-Activated Receptor *α*
PXR: Pregnane X Receptor
TSS: Transcriptional Start Site
